# Supplementary material for: A Zebrafish Model for a Rare Genetic Disease Reveals a Conserved Role for FBXL3 in the Circadian Clock System
Source: Int J Mol Sci. 2022 Feb 21;23(4):2373. doi: 10.3390/ijms23042373 (PMC8875760; doi:10.3390/ijms23042373)
Supplement: Supplementary file 1 [file ijms-23-02373-s001.zip › Figure S1. Zebrafish fbxl3a genomic structure and loss-of-function mutation.pdf]

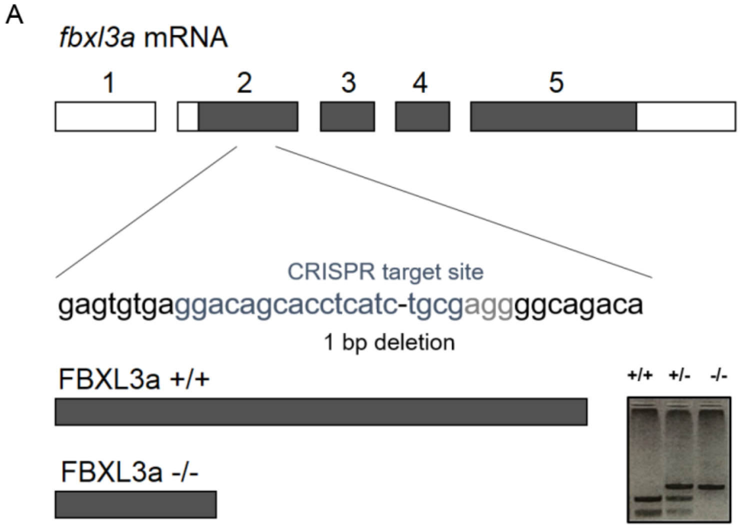

B

```
ttgcagctag tactggttgt gtatgttgag gtaaagttgg ttttatattc  17316517
acacattcta atataatttc agaaaagtat tatttacaat ttgtaaatat  17316467
caaaattata ttagcaataa caatcaaagt acaacattgt tcaaagtcaa  17316417
ataaatagtg ctaatgttgt atcgacgtta tagtcagtga tttcaaaccg  17316367
aatattcaaa gtgacatcgc aaacaatata gggagtatgt cataagttgt  17316317
caccgaacga atctgtgaat ataaaactaa ttttacctta atgttgtgtg  17316267
ttcgtttgtt tatctgtctg catatgtttg tgtgtttgta catttgttaa  17316217
tgtcaatgcg tgtgtgtttg cttgcacctg gtttgtttac actccacatc  17316167
ttgttctcaa cagTTCCCTC AGATCCCCAA AAATGAAAAG GAGGTTGAGC  17316117
AAAGAGTGTG AGGACAGCAC CTCATCATGC GAGGGGCAGA CAGTGTCCTA  17316067
CAAGAGACCG AAACAGCCCT CCGAGGACTT TGGAGATCTG CAGTCCAATT  17316017
GGGTTCGACT TCCCCAGGAG ATCCTGCTTC ACATCTTTCA GTACCTGCCC  17315967
CTTCTTGATC GAGCCTTCGC ATCACAGGTT TGCCATAACT GGAACCATGC  17315917
CTTCCATATG CCAGAGCTGT GGAGATGCTT TGAGTTCGAG CTCAACCAGC  17315867
CGGCCACCTC TTACCTGAAG GCCACGCATC CTGACCTAAT AAAGCAAATA  17315817
ATAAAGAGGC ACTCTAATCA CTTGCAGTAC GTCAGCTTCA AGgtgagagg  17315767
ctcatgttgg aaggtcatgt aatggtcaac aattctggta tgaggaaaaa  17315717
tagaaatgct acttctacta cttatgtggt aggttattgg cagatttgtc  17315667
ttgatttgat tttaaacagt ttacaaatca ggctggcttt taaatggtta  17315617
```

Figure S1. Zebrafish *fbxl3a* genomic structure and loss-of-function mutation. Top, the genomic organization of *fbxl3a* with 5 exons, the first is non-coding. The target sequence in exon 2 and the predicted size of the resulting proteins are depicted. Mutants were identified using PCR amplification followed by enzymatic digestion with *NlaIII*, which recognizes a specific nucleotide sequence within the WT allele (5’-CATG-3’). The mutation is the deletion of the A in this restriction site. The observed *NlaIII*-digested PCR products for WT, heterozygous and homozygous mutant fish are shown (two clear bands of 230 bp and 133 bp are observed for WT, one undigested band of 363 bp for the homozygous mutant and all three bands for the heterozygotes). Bottom, partial *fbxl3a* genomic sequence. Blue letters represent coding sequence within exon 2. The deletion mutation of nucleotide A (red, bold) introduces an early stop codon (red-highlighted) after 80 codons, resulting in a 94 amino acid protein. The sites of PCR primers used for screening are highlighted in yellow. The underlined base sequence represents the *NlaIII* restriction site used in the process of screening. The grey-highlighted nucleotides represent the CRISPR target site.
