# Supplementary material for: A Zebrafish Model for a Rare Genetic Disease Reveals a Conserved Role for FBXL3 in the Circadian Clock System
Source: Int J Mol Sci. 2022 Feb 21;23(4):2373. doi: 10.3390/ijms23042373 (PMC8875760; doi:10.3390/ijms23042373)
Supplement: Supplementary file 1 [file ijms-23-02373-s001.zip › Figure S2. Morphological measurements of zebrafish.pdf]

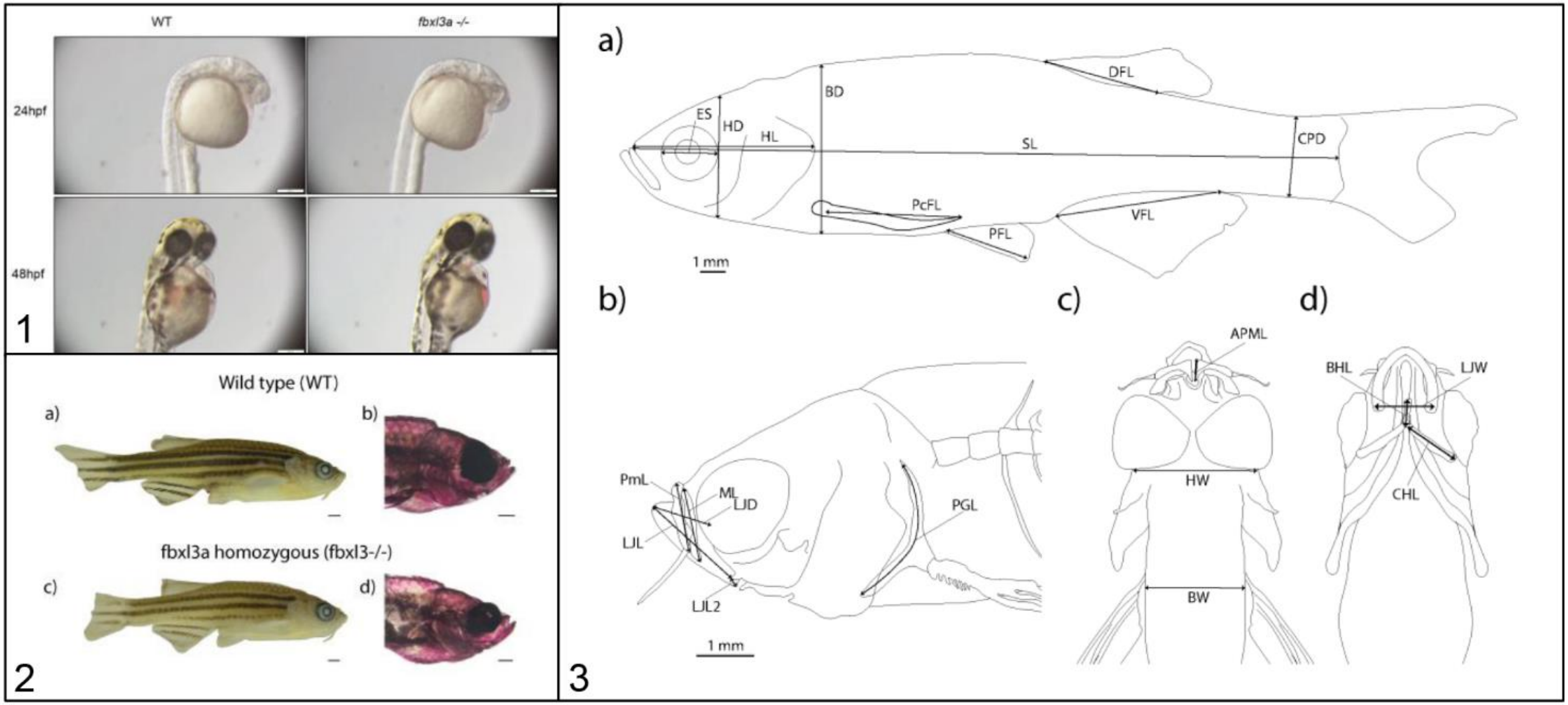

Figure S2. Morphological measurements of zebrafish. (**1**) Morphological measurements of WT and homozygous *fbxl3a* zebrafish embryos. Representative WT and *fbxl3a*$^{-/-}$ embryos, progeny of a cross between heterozygous (*fbxl3a*$^{+/-}$) fish, at the beginning of the pharyngula period (24 hpf) and its end (48 hpf) are shown. The scale bar indicates 200 µm. No evidence for structural abnormalities was detected. (**2**) At the age of 10 weeks, 22 morphological traits were measured. Lateral view of WT (a) and *fbxl3a*$^{-/-}$ (c) fish are presented. Visualization of the skull and pectoral girdle of clear and double-stained WT (b) and *fbxl3a*$^{-/-}$ (d) fish. Staining of the bones (pink) and cartilage (blue) allowed visualization of the skeletal structures. Scale stands for 1 mm. (**3**) Morphometric traits measured to characterize body and viscerocranium dimensions (detailed description of the morphological traits is provided in Table S1). Measurements are superimposed on a line drawing of zebrafish WT in lateral (a-b), ventral (c), and dorsal (d) aspects. No significant evidence for structural abnormalities was detected.
