# Supplementary material for: A Zebrafish Model for a Rare Genetic Disease Reveals a Conserved Role for FBXL3 in the Circadian Clock System
Source: Int J Mol Sci. 2022 Feb 21;23(4):2373. doi: 10.3390/ijms23042373 (PMC8875760; doi:10.3390/ijms23042373)
Supplement: Supplementary file 1 [file ijms-23-02373-s001.zip › Figure S3. Expression of HA-tagged FBXL3a in zebrafish fbxl3a-deficient cells.pdf]

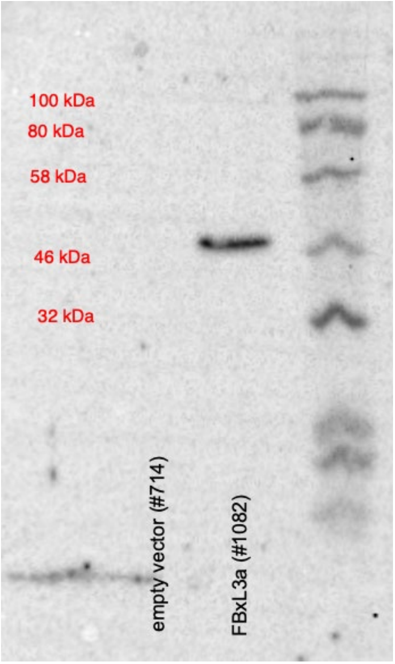

Figure S3. Expression of HA-tagged FBXL3a in zebrafish *fbxl3a*-deficient cells. Western blot analysis, using an anti-HA high affinity rat IgG1 antibody, of cells transfected with HA-tagged expression vector which encodes FBXL3a (ca. 50 kDa) or with an empty expression vector as negative control. On the right-hand side is a lane containing molecular weight markers. The corresponding sizes of the bands are indicated on the left-hand side in kDa.
