# Supplementary material for: A Zebrafish Model for a Rare Genetic Disease Reveals a Conserved Role for FBXL3 in the Circadian Clock System
Source: Int J Mol Sci. 2022 Feb 21;23(4):2373. doi: 10.3390/ijms23042373 (PMC8875760; doi:10.3390/ijms23042373)
Supplement: Supplementary file 1 [file ijms-23-02373-s001.zip › Table S1. Homozygous variants identified by exome sequencing.pdf]

Supplementary Table S1: Homozygous variants identified by exome sequencing

No.	Chromosomal Position	dbSNP	RefSeq	Gene	HGVSc	HGVSp	GERP	CADD score	GnomAD Allele Frequency
1	Chr10:131973139	rs753281821	NM_006541.5	*GLRX3*	c.833A>G	p.Tyr278Cys	1.41	23.7	0.000003979
2	Chr10:135106153	rs202119147	NM_001256617.1	*TUBGCP2*	c.1148C>T	p.Thr383Met	4.87	22.6	0.0003404
3	Chr11:6976932	rs762715444	NM_013250.4	*ZNF215*	c.724A>G	p.Ile242Val	-0.029	8.862	0.00000997
4	Chr11:7712586	-	NM_198185.6	*OVCH2*	c.1553C>T	p.Ser518Phe	5.62	23.7	0
**5**	**Chr13:77581816**	**-**	**NM_012158.4**	***FBXL3***	**c.751C>T**	**p.Arg251Ter**	**5.14**	**37**	**0**
6	Chr14:22938606	-	ENST00000535880.2	*TRDV3*	c.1A>G	p.Met1?	-	21.8	0
