# Supplementary material for: A Zebrafish Model for a Rare Genetic Disease Reveals a Conserved Role for FBXL3 in the Circadian Clock System
Source: Int J Mol Sci. 2022 Feb 21;23(4):2373. doi: 10.3390/ijms23042373 (PMC8875760; doi:10.3390/ijms23042373)
Supplement: Supplementary file 1 [file ijms-23-02373-s001.zip › Text S1. Detailed Clinical evaluation.pdf]

S1 Text. Clinical evaluation

Patient IV-1 is the first child to first cousins Arab-Muslim parents. He was born at term and had a breech presentation. Birth weight was 3.6 Kg (75th percentile) but macrocephaly, already noted at 32 weeks of gestation on prenatal ultrasound, was evident at birth, with head circumference of 37 cm (+3SD). Psychomotor development was always delayed and the parents reported that he started walking at 4 years of age, talking and toilet-training at 6 years of age. Physical examination at 13 years of age revealed macrocephaly (+3SD), short stature (-4SD), myopathic face with persistently open mouth, large protruding ears, "almond shape" eyes and midface hypoplasia. The hair was dry and curly. The first toes and fingers were slightly broad and the feet were flat. There was generalized muscle hypotonia with attenuated deep tendon reflex. The patient was clumsy and had poor coordination but could jump and climb up stairs. He had a satisfactory eye contact but this was constantly interrupted because of his very short attention span. He talked spontaneously but in short sentences and had difficulties in pronunciations. He could not name colors and had no comprehension of size and numbers. Overall, his cognitive level was assessed at a 26 months level (WISC-R test and Baylor test). Brain MRI was normal, as were ophthalmologic examination, hearing test and skeletal survey.

His sister (patient IV-3), who was 11 years old at examination, had similar manifestations with prenatal macrocephaly, muscle hypotonia since birth with delayed psychomotor development thereafter and short stature. Facial appearance was very similar to her brother. At 11 years her height was -3SD but bone age and endocrinological evaluation were normal for her age. As her brother, she started walking at 4 years of age, talking and toilet-training at 6 years of age.

The pregnancy of the fifth child in the family (patient IV-5) was monitored closely. Macrocephaly and midfacial hypoplasia (present in both affected patients) were already noted at 13 weeks of gestation. Macrocephaly with head circumference of 37.8 cm and marked muscle hypotonia were present at birth. On examination at one year of age, head circumference was >98th percentile, weight and height were both on the 75th percentiles. Muscle hypotonia was prominent and the child could not roll over or be seated without support. All children attended the same special education school.

Laboratory investigation, performed in patients IV-1 and IV-3, including blood creatine kinase and amino acids, urinary organic acids and chromosomal analysis were normal, and fragile X excluded. MPS and oligosaccharides testing in urine were normal.
